# Hochschulübergreifende Digitale Lehr‑/Lernkonzepte zum Geschäftsprozessmanagement – Herausforderungen und Lessons Learned

**DOI:** 10.1365/s40702-021-00802-3

**Published:** 2021-09-29

**Authors:** Ralf Plattfaut, Armin Stein, Katrin Bergener

**Affiliations:** 1 Process Innovation and Automation Lab, Fachhochschule Südwestfalen, Lübecker Ring 2, 59494 Soest, Deutschland; 2 Institut für Wirtschaftsinformatik/European Research Center for Information Systems, Westfälische Wilhelms-Universität Münster, Leonardo-Campus 3, 48149 Münster, Deutschland

**Keywords:** Hochschulübergreifende Lehre, Hochschullehre, E‑Learning, Distance Learning, E‑Klausur, Cross-University Teaching, E‑Learning, Distance Learning, Electronic Examination

## Abstract

Durch die Covid-19-Pandemie und die entsprechenden „Lock-Downs“ wurde der digitale Lehrbetrieb an Hochschulen weiter in den Vordergrund gerückt. Die in den vergangenen Jahren und Jahrzehnten gesammelten Erfahrungen im E‑Learning und Blended Learning-Bereich sind zwar hilfreich, für die jetzt geforderte rein digitale Lehre in den meisten Fällen aber noch unzureichend.

Im Wintersemester 2020/2021 haben die Westfälische Wilhelms-Universität Münster und die Fachhochschule Südwestfalen eine Online-Lehrveranstaltung zum Thema „Fundamentals of Business Process Management“ im Rahmen der jeweiligen Bachelorstudiengänge *Wirtschaftsinformatik* durchgeführt. Die Online-Vorlesung selbst diente als Vorbereitung auf eine darauf aufbauende seminaristisch angelegte internationale Winter School. Die Inhalte basierten auf einem anerkannten und gleichnamigen Lehrbuch, für die Online-Vorlesung wurde auf entsprechendes von den Lehrbuchautor*innen vorbereitetes und durch die Dozent*innen kuratiertes Videomaterial zurückgegriffen. Während an der Fachhochschule regelmäßige virtuelle Austauschrunden (Video-Konferenzen) angeboten wurden, um potenzielle Fragen der Studierenden zu beantworten, war der Vorbereitungskurs an der Universität als reine Online-Veranstaltung ausgelegt. Die Prüfung wurde an beiden Hochschulen als Open Book Online-Klausur über die jeweilige Lehrplattform abgelegt. Die entsprechenden Aufgaben wurden von den Dozent*innen arbeitsteilig zuerst auf der eigenen Lehrplattform (Moodle) erstellt und dann in die jeweils andere Lehrplattform übertragen.

In diesem Artikel berichten wir von diesem hochschulübergreifend durchgeführten Modul und explizieren aufbauend auf den Erfahrungen der Dozent*innen sowie dem Feedback der Studierenden Herausforderungen und Lessons Learned. Wir diskutieren Möglichkeiten zur Weiterentwicklung und geben Hinweise für die zukünftige Gestaltung entsprechender Kurse.

## Abstract (en)

The Covid 19 pandemic and the corresponding “lock-downs” have brought digital teaching at universities further to the fore. The experience gained over the past years and decades in e‑learning and blended learning by higher education institutions is helpful, but in most cases still insufficient for the purely digital teaching that is now required. During the winter term 2020/2021, the University of Münster and the South Westphalia University of Applied Sciences held an online course on “Fundamentals of Business Process Management” as part of their respective bachelor’s degree programmes in *Wirtschaftsinformatik* (Information Systems). The lecture served as preparation for an international Winter School on the topic of the same name. The content was based on a recognised textbook. For the online lecture, corresponding video material prepared by the authors of the textbook and curated by the lecturers was used. While regular virtual feedback rounds (video conferences) were offered at the University of Applied Sciences to answer potential questions of the students, the course at the university was designed as purely online. The examination was taken at both institutions as an open book online exam via the respective teaching platform. The corresponding tasks were created by the lecturers in a division of labour, first on their own teaching platform and then transferred to the other.

In this article, we report on this teaching module, which was carried out across the universities. Building on the experiences of the lecturers and the feedback from the students, we explain the challenges and lessons learned. We discuss possibilities for further development and give hints for the future design of corresponding courses.

## Einführung und Motivation

Forschung zum „digitalen Klassenzimmer“, E‑Learning, Blended Learning oder virtuellem Lehren und Lernen gibt es in unterschiedlichen Fachdisziplinen (d. h. Erziehungswissenschaften, Kommunikationswissenschaften, Informatik) schon seit den 1980er Jahren (Hiltz 1986; Palloff und Pratt 2013). Seitdem hat sich sowohl die Forschung als auch die Praxis weiterentwickelt. So konnte in den vergangenen Jahrzenten vermehrt Erfahrung mit digitalen Konzepten wie beispielsweise virtuellen Seminaren (Bergener et al. 2012) gesammelt werden. Nichtsdestotrotz stellt die digitale Lehre, die seit Beginn der Covid-19-Pandemie unabdingbar für alle Lehrenden zur Pflicht wurde, Lehrende sowie Studierende vor Herausforderungen. Vor der Pandemie wurde an deutschen Universitäten und Fachhochschulen in überwiegendem Maße in Präsenz gelehrt und die Vorteile des Präsenzunterrichts (feste Zeiten, d. h. Struktur im Alltag, persönlicher Kontakt zu Dozent*innen und Kommiliton*innen) gegenüber dem Fernunterricht (Anonymität) betont. Die Monate der Pandemie haben gezeigt, dass digitale Lehre funktionieren kann und neben den Herausforderungen auch Vorteile bietet. Durch die zum Teil zeitversetzte Lehre (aufgezeichnetes Video vs. synchrone Vorlesung) wandelt sich die Rolle der Dozent*innen mehr und mehr zu Lernbegleiter*innen.

Gleichzeitig sind insbesondere Grundlagenveranstaltungen in Bachelorstudiengängen sowohl hochschulübergreifend als auch hochschulformübergreifend (d. h. zwischen Fachhochschulen und Universitäten) ähnlich und greifen zum Teil auch auf dieselbe Grundlagenliteratur zurück. Auch die Lernziele, d. h. die nachprüfbar vorhandenen und erwünschten Ergebnisse, die am Ende eines Lernprozesses stehen (Terhart 2005), sind in diesen Grundlagenveranstaltungen unterschiedlicher Hochschulformen häufig die gleichen. Als Vorbereitung auf eine digitale internationale Winter School zum Thema Business Process Management (BPM) bestand die Herausforderung, in unterschiedlichen Studiengängen der Wirtschaftsinformatik an einer Fachhochschule und einer Universität eine Lehrveranstaltung zum Thema „Fundamentals of Business Process Management“ anzubieten. Die Studierenden beider Institutionen sollten im Rahmen dieser Lehrveranstaltung die Grundlagen von BPM vermittelt bekommen, um dann im Rahmen der daran anschließenden Winter School vertiefte Kenntnisse in ausgewählten Themenbereichen zu erhalten. Dazu wurde auf ein gleichnamiges Lehrbuch (Dumas et al. 2018) und entsprechendes von den Autoren vorbereitetes Videomaterial (Mendling 2020) zurückgegriffen. Die Dozent*innen haben als Lernbegleitung das Videomaterial kuratiert und den Studierenden über die hochschulspezifischen Lehrplattformen zur Verfügung gestellt. Während an der Fachhochschule regelmäßige virtuelle Austauschrunden (Video-Konferenzen) angeboten wurden, um potenzielle Fragen der Studierenden zu beantworten (ein „Push“-Konzept), war der Kurs an der Universität als reines Onlinestudium ausgelegt, wo es lediglich eine Fragestunde bei Bedarf vor der Klausur gab („Pull“-Konzept). Die Prüfung wurde an beiden Hochschulen als Open Book Online-Klausur über die jeweilige Lehrplattform abgelegt. Aspekte von BPM werden im Rahmen eines Wirtschaftsinformatikstudiums auch in anderen Lehrveranstaltungen behandelt, daher ging es in der Vorbereitung und Durchführung dieser Lehrveranstaltung konkret darum, einen gleichen Lernstand der Studierenden beider Institutionen sicherzustellen, sowie eine gemeinsame Prüfung durchzuführen.

In diesem Artikel berichten wir von diesem Fallbeispiel als hochschulformübergreifende Kooperation (Details in Abschn. 2), den Erfahrungen der Dozent*innen (Abschn. 3) und Studierenden (Abschn. 4) sowie den dabei auftretenden Herausforderungen (Abschn. 5). Darauf aufbauend leiten wir Lessons Learned ab (Abschn. 6) und schließen mit einer kurzen Zusammenfassung und Ausblick (Abschn. 7).

## Fallbeispiel: Hochschulübergreifender Kurs zum Geschäftsprozessmanagement

Seit 2012 wird in jedem Wintersemester ein internationaler, englischsprachiger einwöchiger Bachelor-Kurs „BPM (Business Process Management) Winter School“ von der Universität Liechtenstein sowie der Universität Münster als ausrichtende Partner angeboten. Im Schnitt nahmen Studierende von sechs Universitäten – hauptsächlich aus dem europäischen Ausland, aber auch aus Südkorea und Australien – jährlich an der Winter School teil. Ziele dieser Veranstaltung waren und sind, internationalen Bachelor-Studierenden für eine Woche im seminaristischen, englischsprachigen Unterricht vor Ort die Grundlagen des Geschäftsprozessmanagements darzustellen, den Studierenden die Möglichkeit zu bieten, ein erstes internationales Netzwerk aufzubauen, die Anwendung der im Kurs vermittelten Inhalte in der Praxis durch das Einbeziehen und Besuchen von Unternehmen zu vermitteln, sowie ein mögliches Master-Studium im Bereich Geschäftsprozessmanagement näher zu bringen.

Bei den Inhalten wurde sich in der Regel an dem *Handbook of Business Process Management* (vom Brocke und Rosemann 2015) orientiert, spezifischer an den *Six Core Elements of Business Process Management* (vom Brocke und Rosemann 2015, pp. 105–122). Diese betrachten eine Unternehmung mit Blick auf das Geschäftsprozessmanagement aus den Perspektiven *Strategic Alignment, Governance, Methods, Information Technology, People*, und *Culture*. Im Kurs wurden diese Bereiche angesprochen und den Studierenden durch Vorlesungen und Übungen nähergebracht. Der Lernerfolg wurde mit einer herkömmlichen sechzigminütigen Klausur am Ende der Woche überprüft. Die Vorlesungen selbst wurden durch Dozent*innen der Institute für Wirtschaftsinformatik der Universitäten Liechtenstein und Münster durchgeführt. Insgesamt wurde die Winter School in der Vergangenheit mit drei ECTS (European Credit Transfer and Accumulation System, European Commission (2021)) bewertet, was einem erwarteten Arbeitsaufwand von ca. 75 h entspricht.

Die Einbettung in die lokalen Curricula erfolgt und erfolgte flexibel – so ist die Veranstaltung an der Universität Münster Teil eines sogenannten Vertiefungsmodules, das aus 6 ECTS Seminar und 3 ECTS Vorlesung und Klausur besteht (Institut für Wirtschaftsinformatik 2019). Der Seminarteil wurde durch die Studierenden zwischen Oktober und Februar an der Heimatuniversität erbracht und diente der Vorbereitung auf die Veranstaltung in Liechtenstein.

Durch die Pandemie war eine Durchführung des Kurses in der bewährten Form einerseits durch die eingeschränkten Reisemöglichkeiten, andererseits aber auch durch die verpflichtende Virtualisierung der Lehre an allen beteiligten Hochschulen zu dem Zeitpunkt nicht möglich – die oben angeführten Ziele blieben jedoch erhalten. Durch den langen zeitlichen Vorlauf bis Februar 2021 konnte das Konzept jedoch entsprechend umgestellt werden. Kern war hierbei, dass der Vorlesungsteil, der ursprünglich vor Ort in Liechtenstein stattfand, dezentral vorgezogen wird und die eigentliche Veranstaltung die Stellung eines Seminars einnahm. Die Inhalte des *Handbook of Business Process Management*, konkret die sechs Kernelemente, waren somit nicht mehr Gegenstand der eigentlichen Winter School, was die Lehrenden der Universität Liechtenstein entlastete. Der virtuelle Ansatz sollte die Möglichkeit schaffen, vertiefende und spezifischere Inhalte der aktuellen Geschäftsprozessmanagement-Forschung zu vermitteln und zur Diskussion zu stellen. Durch das Online-Format der Winter School konnten Dozent*innen verschiedener internationaler Hochschulen eingeladen werden, über ihre Schwerpunkte des Geschäftsprozessmanagements zu referieren. Von den an der Winter School teilnehmenden Studierenden wurde erwartet, dass sie sich selbständig mit den Grundlagen vertraut machen. Sowohl die Universität Münster also auch die Fachhochschule Südwestfalen entschieden sich dazu, ihre Studierenden eine Online-Vorlesung (Mendling 2020) verfolgen zu lassen und den Lernerfolg durch eine Klausur zu überprüfen.

Insgesamt vergab die Fachhochschule Südwestfalen für dieses Modul 6 ECTS, konkret 3 ECTS für den Online-Kurs und die zugehörige Klausur, sowie 3 ECTS für die BPM Winter School (Anrechnung als Current Developments in Business IT, Fachhochschule Südwestfalen (2018)). Die Universität Münster bewertete diese Prüfungsteile gleich, erlegte den Studierenden aber einen weiteren Seminarteil zum Thema Geschäftsprozessmanagement auf, in dem sie jeweils über unterschiedliche Fallbeispiele aus vom Brocke und Mendling (2018) referieren mussten. Dieser Teil wurde ebenfalls mit 3 ECTS bewertet.

Der vorliegende Beitrag konzentriert sich auf die vorgezogene Online-Vorlesung und die dazugehörige Klausur. Die Online-Vorlesung von Mendling (2020) diente dafür als Grundlage. Die Dozent*innen der FH Südwestfalen und der Universität Münster kuratierten eine eigene Liste, die um einige wenige zusätzliche Videos ergänzt wurde (Stein und Plattfaut 2020).

Da sowohl die Universität Münster als auch die FH Südwestfalen die Lernplattform *Moodle* (https://moodle.org) verwenden, die auch für die Durchführung von Online-Prüfungen genutzt wird, wurde die Idee realisiert, die gleiche Klausur in beiden Systemen zu nutzen. Durch die Standardisierung der Schnittstellen des Open Source-Systems konnten beide Hochschulen auf den gleichen Fragensatz zurückgreifen.

## Erfahrungen der Dozent*innen

Aus Sicht der Dozent*innen hat die digitale, hochschulübergreifende Lehre gut funktioniert. Aufgrund der Einigung, das Grundlagenwissen unter Rückgriff auf ein Standardwerk (Dumas et al. 2018) zu vermitteln, gestaltete sich die Definition von Kapiteln als einfach. Dadurch, dass das verwendete Standardwerk von den Autoren sowohl mit beispielhaften Aufgaben mit Musterlösungen versehen als auch begleitende Foliensätze sowie Lehrvideos bereitgestellt wurde, lag der Fokus auf der gewissenhaften Kuratierung des Materials. Hier wurden in drei Videokonferenzen Schwerpunkte diskutiert und eine Youtube-Playlist für die Studierenden erstellt (Stein und Plattfaut 2020). Diese enthält das Material, welches von den Dozent*innen als geeignet für die Erreichung der Lernziele eingeschätzt wurde. Die Youtube-Playlist wurde zusammen mit einem Zugang zum Buch sowie einführenden Texten auf den jeweiligen Lernplattformen (in beiden Fällen Moodle) zur Verfügung gestellt. Das gemeinsame (verteilte) Arbeiten auf der Youtube-Plattform stellte sich hierbei als ebenso unproblematisch heraus, wie das Teilen des Bildschirms, sodass ein*e Dozent*in aktiv, ein*e passiv mitwirken konnte. Als gute Unterstützung für die geteilte Lösung hat sich auch die Kommentierungsfunktion von Zoom erwiesen, mit der die/der passive Teilnehmer*in unkompliziert auf zu besprechende Bereiche hinweisen konnte.

Während der Kursdurchführung wurde an beiden Hochschulen ein unterschiedliches Modell durchgeführt. An der WWU Münster wurden die Studierenden angehalten, sich den Lehrinhalt selbstständig und im eigenen Tempo zu erarbeiten. Fragen konnten in einem Diskussionsforum auf der Lernplattform Moodle gestellt und mit dem Dozenten diskutiert werden. Allerdings sind in der Vorbereitung auf die Klausur keine Fragen aufgekommen. Von einer optional angebotenen Fragestunde vor der Klausur wurde kein Gebrauch gemacht. An der FH Südwestfalen wurde der Kursinhalt nochmals logisch in fünf Abschnitte unterteilt, war aber in Summe inhaltlich identisch. In jedem Abschnitt mussten sich die Studierenden den Lerninhalt wie an der WWU Münster selbstständig erarbeiten. Nach einem Abschnittsende fand eine Videokonferenz über Zoom statt, in der die Studierenden Fragen zum Lerninhalt stellen und gemeinsam mit dem Dozenten diskutieren konnten. Hierdurch sollten gegebenenfalls vorliegende mangelnde Vorkenntnisse der Studierenden aufgegriffen werden. Allerdings wurden nur in vier der fünf Videokonferenzen Fragen gestellt, die sich sowohl auf Inhalte der Lernvideos als auch auf Inhalte aus dem Lehrbuch bezogen. Ob die in Summe wenigen Rückfragen der Studierenden auf die Qualität des Buch- und Videomaterials zurückzuführen ist, oder eine generelle Beobachtung zum selbständigen Online-Lernen ist, kann nicht beantwortet werden. Die Studierenden begriffen die Videokonferenzen auf jeden Fall als zusätzliches, freiwilliges Lehrangebot.

Insgesamt konnten die Dozent*innen (auch über diesen Kurs hinaus) beobachten, dass durch die Onlinelehre weniger Interaktion und Diskussion mit den Studierenden und insgesamt der Studierenden untereinander stattfand. Im Rahmen von Videokonferenzen ergaben sich nur sehr selten Diskussionen, die sich in einer Präsenzlehre in Seminarveranstaltungen häufiger ergeben bzw. auch besser stimuliert werden können. Dies war aus Sicht der Autor*innen auch darauf zurückzuführen, dass die Studierenden in der Regel die Kamera ausgeschaltet ließen und es damit auch keine nonverbalen Kommunikationssignale gab, auf die reagiert werden konnte.

Für die Prüfung wurde auf die Prüfungsform einer online-basierten Open Book-Klausur zurückgegriffen. Diese Klausurform erlaubt die Prüfung komplett in den jeweiligen Moodle-Plattformen der beiden Hochschulen. Die Studierenden können dabei das Lehrbuch verwenden oder auf die Videos zurückgreifen. Für die Klausuraufgaben haben die Dozent*innen von beiden Hochschulen Klausuraufgaben erstellt und unter Nutzung der Export-Import-Funktion von Moodle in einem Fragepool zusammengeführt. Aufgrund der Verwendung derselben Lerninhalte in beiden Hochschulen konnte eine identische Prüfung abgenommen werden. Dabei wurden verschiedene Fragetypen verwenden (Multiple-Choice, Rechenaufgaben mit Zufallszahlen, Zuordnungsaufgaben, offene Textaufgaben) und verschiedene Lernziele nach Bloom (1956) geprüft. So wurden beispielsweise durch einfache Wahr‑/Falsch-Aufgaben Faktenwissen abgefragt. Durch offene Textaufgaben mussten Sachverhalte analysiert und entsprechendes Wissen angewendet werden. Außerdem wurden modellierte Geschäftsprozesse präsentiert, die von den Studierenden evaluiert werden mussten. Der Austausch der Klausuraufgaben gestaltete sich weitgehend problemlos (zu konkreten Lessons Learned siehe auch Abschn. 6). Der Aufwand für die Vorbereitung dieser Art der (gemeinsamen) Prüfung war lediglich durch das Austesten der Funktionalitäten einmalig erhöht. Das Konzipieren der Klausur ging auch über die virtuelle Kommunikation problemlos. Gefühlt macht die begrenzte Verfügbarkeit von Aufgabentypen in Moodle die Auswahlentscheidungen und die Definition des Erwartungshorizontes pro Aufgabe sogar einfacher.

Aus Sicht der Dozent*innen entsprechen die Ergebnisse der Klausur einem zu erwarteten Rahmen. Der Notenspiegel schöpft fast die gesamte Bandbreite aus und reicht von 1,3 bis 5,0, wobei es auch einige Studierende gab, die diesen Teil des Gesamtmoduls nicht bestanden haben. Während der Durchführung der Klausur, während der die Studierenden auf einen Helpdesk mittels Zoom zugreifen konnten, gab es keine Auffälligkeiten. Aufgrund der kleinen Anzahl an Beobachtungen sehen wir davon ab, die unterschiedliche Leistung der Fachhochschul- und Universitätsstudierenden zu diskutieren, da andere belastende Einflüsse (wie bspw. parallel fällige Abgaben, Klausuren, etc.) nicht berücksichtigt wurden.

## Erfahrungen der Studierenden

Aus Sicht der Studierenden hat die rein digitale und selbstverantwortliche Lehre nicht die persönliche Interaktion in einer klassischen Lehrveranstaltung ersetzt. In einer nach der Veranstaltung durchgeführten kurzen Umfrage gaben die Studierenden an, dass sie sich durch den Online-Kurs gut auf die Klausur vorbereitet fühlten (siehe Tab. 1; von den in Summe 13 teilnehmenden Studierenden haben 7 Rückantworten gegeben, davon 3 von der WWU Münster und 4 von der FH Südwestfalen). Es wurde aber auch deutlich, dass das dem Kurs zugrundeliegende Buch als zusätzliches Nachschlagewerk zu den Videos notwendig war. Bei der Bewertung gab es keine Unterschiede zwischen den Studierenden der WWU Münster und der FH Südwestfalen. Auf die Frage, ob die Anleitungen der Dozent*innen gut geeignet waren, um durch die Lehrmaterialien zu führen, haben die Studierenden der FH Südwestfalen tendenziell etwas positiver geantwortet, insbesondere wurden die durchgeführten Videokonferenzen gelobt. Von den Studierenden der Universität Münster wurde das Fehlen von zusätzlich proaktiv angebotenen synchronen Gesprächen nicht kritisiert, sodass davon ausgegangen werden kann, dass dies nicht vermisst wurde. In Bezug auf das Erwartungsmanagement wurde kritisiert, dass der Kurs nicht die Themen der Winter School selbst behandelt, was aber einem Verständnisproblem des Studierenden zugeordnet wird, da dieser Sachverhalt initial deutlich betont wurde. Bezogen auf die Passgenauigkeit der Prüfung zu den angebotenen Lehrmaterialien (Lehrbuch und Lehrvideos) haben sich die Studierenden ebenfalls passend geprüft gefühlt. Hier gab es keine Auffälligkeiten in der durchgeführten Umfrage, lediglich die Form der Online-Prüfung wurde durch zwei Studierende in Bezug auf die gefühlt starre Form, die durch Moodle umgesetzt wird, kritisiert. Schließlich wurde die allgemeine Betreuung von den Studierenden beider Institutionen überwiegend positiv bewertet.

**Tab. 1:** Auswertung der Studierendenbefragung

Fragen (übersetzt)	--	–	0	+	++	Beispielhafte Freitextantworten
	# Antworten					
1. Wie gut hat Sie der videogestützte Online-Kurs auf die Klausur vorbereitet?	0	1	1	3	2	“The online videos were good and well introduced. However, they were not enough for my exam preparation I always had to go back to the book for more explanation.”
2. Wie gut hat Sie der videogestützte Online-Kurs auf die Winter School vorbereitet?	0	0	2	2	3	“Not all the video courses covered the topics given at the winter school, however I felt that I had initial knowledge and I felt prepared for the seminars.”
3. Wie gut hat die Klausur den Inhalt des Kurses abgedeckt?	0	0	3	3	1	“The exam evaluated the video course quite well, but it also felt like a trial-and-error situation.”
4. Wie gut haben die Dozent*innen Sie während des Online-Kurses begleitet und angeleitet?	0	1	1	3	2	“Everything was well explained before courses/exam, the whole process was very easy.”

Legende: -‑ “überhaupt nicht”, – “nicht genügend”, 0 “teilweise”, + “eher gut”, ++ “gut”

Während der im Anschluss stattfindenden Winter School haben die Studierenden in einer Feedback-Session und in individuellen Gesprächen während der Pausen ebenfalls positives Feedback geäußert. Der Online-Kurs erlaubte gerade durch die stets verfügbaren Videomaterialien eine Wiederholung der Themen nach eigenen Wünschen und in eigener Geschwindigkeit. Inhaltlich wurde durch die Online-Lehre und durch die anschließende online-basierte Klausur eine gute Grundlage für die Diskussionen während der seminaristisch ausgelegten Winter School gelegt. Hier haben die Studierenden geäußert, dass sie sich in Vorbereitung auf einzelne Teile der Winter School erneut mit den Lehrmaterialien beschäftigt haben und entsprechende Videos erneut geschaut haben. Dies hat dann aber nicht fokussiert als „Lernen“ stattgefunden, sondern begleitend zu sonstigen Tätigkeiten im Haushalt mit dem Ziel einer „Auffrischung“. Die Vermittlung der Grundlagen mittels eines Online-Kurses, die im Anschluss durch interaktive Veranstaltungen vertieft werden, scheint sich hier bewährt zu haben.

## Herausforderungen

Bei der Durchführung des hochschulübergreifenden Online-Kurses zum Geschäftsprozessmanagement traten drei Haupt-Herausforderungen auf, die sich alle auf die Prüfungen bezogen: Die Neuheit der Prüfungsform vor dem Hintergrund der Corona-Situation und den geänderten Prüfungsformen im Allgemeinen; die Prüfungsform der online-basierten Open Book-Klausur im Besonderen; und die Terminierung der Prüfungsleistungen. Diese drei Herausforderungen bauen aufeinander auf und werden im Folgenden diskutiert.

1. *Online-Prüfung:* Vor der Corona-Pandemie waren in den entsprechenden Studiengängen an beiden Hochschulen keine Online-Prüfungen erlaubt. In dieser Situation hätte eine Präsenzprüfung mit denselben oder ähnlichen Aufgaben durchgeführt werden können. Durch das Online-Gebot und die Änderungen der Prüfungsordnungen konnte die Prüfung als online-basierte Open Book-Klausur durchgeführt werden. Hier war es von Vorteil, dass die Prüfungsordnungen in beiden Studiengängen ähnlich angepasst wurden, so dass grundsätzlich dasselbe Format möglich war.
2. *Open Book-Klausur:* In einer Open Book-Klausur können die Studierenden während der Klausur auf die Lernmaterialien zurückgreifen. Hierdurch wird das Abfragen von Faktenwissen und damit der untersten Ordnungsstufe der Lernzieltaxonomie nach Bloom (1956) für die Studierenden stark vereinfacht. Ein simples Nachschlagen kann aber durch einen Zeitdruck erschwert werden. Diese Zeitknappheit muss hinterher bei der Bewertung der Klausuren und dem Anlegen eines Notenrasters berücksichtigt werden. Darüber hinaus besteht bei einer online-basierten Klausur die Gefahr, dass Studierende untereinander kommunizieren, was beispielsweise bei einem Austausch von Lösungen einen Täuschungsversuch darstellen würde. Allerdings lässt sich dies in einer online-basierten Klausur nur schwer nachweisen. Aus diesem Grund wurde die Klausur so konstruiert, dass Fragen aus einem Fragenpool gezogen oder bei Rechenaufgaben auf Zufallszahlen zurückgegriffen wurden. Ein Beispiel dafür ist der einfache Austausch des Kontexts (z. B. Vor- und Nachteile einer Methode zur Prozessanalyse am Beispiel eines Taxiunternehmens oder einer Pizzabäckerei). Außerdem konnten bei Multiple-Choice-Aufgaben die Antwortmöglichkeiten durchmischt werden, so dass die Antworten in unterschiedlicher Reihenfolge vorlagen. Dies führte dazu, dass Studierende gleichwertige aber inhaltlich unterschiedliche Klausuren erhielten.
3. *Klausurtermin:* Die dritte Herausforderung bestand in der Terminierung der Prüfungsleistung. Hier mussten die Klausuren im selben Zeitfenster geschrieben werden, um eine Rekonstruktion des Fragepools zwischen zwei Klausurterminen zu vermeiden. Im hier beschriebenen Fall verständigten sich die Dozent*innen darauf, die Klausur in beiden Klausurzeiträumen gleich zu terminieren und durchzuführen.

## Lessons Learned

**Abb. 1:**
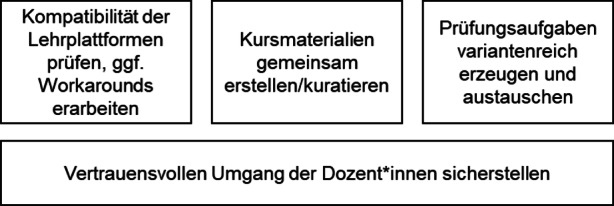
Lessons Learned

Aufbauend auf den Herausforderungen leiten wir vier Lessons Learned ab. Diese werden im Folgenden kurz diskutiert und sind in Abb. 1 grafisch dargestellt.

Zuerst war es wichtig, die *Kompatibilität der technischen Basis* für die digitale Lehre zu schaffen. Im geschilderten Fall bezog sich das hauptsächlich auf die eingesetzte Lernplattform, die in beiden Fällen Moodle in einer ähnlichen Version war. Hierdurch war der Austausch von Lehr- und Prüfungsmaterialien über Ex- und Importfunktionalitäten leicht möglich. Wären die Lernplattformen nicht direkt kompatibel, hätten andere Dateiformate zum Austausch eruiert werden müssen (beispielsweise können in Moodle auch Prüfungsaufgaben aus anderen Programmen und Plattformen wie WebCT oder ExamView importiert werden, ein Export in die entsprechenden Dateiformate ist aber nicht ohne weiteres möglich). Hier bietet es sich grundsätzlich für Dozent*innen an, schon früh in der Planung von hochschulübergreifenden onlinegestützten Lehrveranstaltungen die Kompatibilität der Lehrplattformen zu prüfen und ggf. Workarounds zu ermöglichen. Die technische Basis wurde bereits im September 2020, also vier Monate vor der eigentlichen Klausur geprüft, was die Umsetzung erleichterte, da sich auf die Planung der Inhalte konzentriert werden konnte.

Anschließend mussten sich die Dozent*innen von beiden Hochschulen auf dieselben Kursmaterialien einigen. Hierbei war es von Vorteil, dass an beiden Hochschulen schon dasselbe Lehrbuch verwendet wurde. Trotzdem konnte weder das Lehrbuch noch die bereitgestellten Videos vollumfänglich übernommen werden. Stattdessen musste im Austausch zwischen den Dozent*innen eine *Kuratierung der Lehrmaterialien* stattfinden. Hier wurden durch Austausch der Dozent*innen Schwerpunkte gesetzt und andere Lehrinhalte nicht in voller Tiefe behandelt und entsprechend depriorisiert. Gerade diese kollegiale Schwerpunktsetzung erfordert ein gewisses gegenseitiges Vertrauen (siehe auch unten).

Aufbauend auf den beiden ersten Lessons Learned war dann (drittens) der Austausch der Prüfungsaufgaben zwischen den Hochschulen problemlos möglich. Hierbei war es nur notwendig, sich auf operative Standards bei der Fragenerstellung zu einigen. Beispielsweise war es sinnvoll, die Fragen in der Benennung kapitelweise zu nummerieren, um auch nach Ex- und Import eine Sortierung zu ermöglichen. Im Fall von deutschsprachigen Prüfungen wäre auch eine Anrede der Studierenden abzusprechen („Duzen“ versus „Siezen“). Außerdem ist es empfehlenswert, testweise *unterschiedliche Fragetypen zu verwenden und auszutauschen*. Moodle erlaubt es, komplexere Fragetypen per Plugin auf den entsprechenden Installationen zur Verfügung zu stellen. Hierdurch können trotz prinzipieller Kompatibilität der Plattform Probleme beim Austausch der Aufgaben auftreten.

Im hier dargestellten Kontext der Covid-19-getriebenen Umstellung der Veranstaltung in ein digitales Setting war abschließend das *Vertrauen zwischen den Lehrenden* von fundamentaler Bedeutung. Zum einen war bei der initialen Einschätzung der Qualität der Lehrvideos ein Grundvertrauen der Dozent*innen in die Autoren des Lehrbuchs (und damit auch in die Ersteller der Lehrvideos) vorhanden. Zum anderen ist die Kuratierung der Lehrmaterialien und die verteilte Erstellung der Klausuraufgaben sicherlich in einem vertrauensvollen Kontext einfacher. Die Dozent*innen konnten auf gemeinsame Vorerfahrungen zurückgreifen und haben sich so weitgehend auf die Qualität der Aufgaben des jeweils anderen verlassen.

## Zusammenfassung und Ausblick

Im Wintersemester 2020/2021 wurde eine gemeinsame hochschulformübergreifende digitale Lehrveranstaltung zum Thema „Fundamentals of Business Process Management“ im Rahmen der jeweiligen Bachelorstudiengänge Wirtschaftsinformatik an der Fachhochschule Südwestfalen und der Westfälischen Wilhelms-Universität Münster durchgeführt. Es hat sich gezeigt, dass eine gemeinsame Kuratierung der Lehrmaterialien (Mendling 2020), die Bereitstellung der Materialien auf den hochschulspezifischen Plattformen sowie eine parallel durchgeführte Open Book Online-Klausur über Moodle eine gute Möglichkeit ist, um gleiche Eingangsvoraussetzungen für Studierende unterschiedlicher Institutionen zu schaffen. Dies bildet eine gute Grundlage und einen vergleichbaren Wissensstand für die anschließende vertiefende Seminarwoche. Hinzu kommt, dass sich ein gemeinsamer Online-Kurs insbesondere dafür eignet, den Wissensstand von Studierenden unterschiedlicher Institutionen anzugleichen, da er vor Präsenzveranstaltungen flexibel eingeplant werden kann. Dies ermöglicht eine individuelle Auseinandersetzung mit den Inhalten sowie eine maximale zeitliche Flexibilität für die Studierenden. Die Erfahrungen auf Studierenden- und auf Dozent*innenseite waren durchweg positiv und Überlegungen gehen dahin, diese virtuellen Lehr‑, Lern-, und Prüfformate auch nach der Pandemie beizubehalten.

Auch wenn die Online-Durchführung der Lehrveranstaltung im Wintersemester 2020/2021 aufgrund der Covid-19-Pandemie eine Ad-hoc-Lösung darstellte, lassen sich mehrere Möglichkeiten der Weiternutzung und -entwicklung festhalten. Zum einen ist die Einbindung weiterer Hochschulen in dieses Lehrformat denkbar. Die aktuell beteiligten Institutionen verwenden beide die Lernplattform Moodle. Dadurch konnten Prüfungsfragen über Schnittstellen ausgetauscht werden und die Online-Prüfung parallel in beiden Systemen durchgeführt werden. Weitere Export-Formate in Moodle bieten die Möglichkeit, Daten auch mit anderen Lernplattformen (z. B. ILIAS, Stud.IP) auszutauschen. Die Open-Source-Lern- und Bildungsplattformen ILIAS, Moodle und Stud.IP werden zusammen an über 90 % der deutschen Hochschulen eingesetzt. Die Möglichkeit eines Austauschs von Prüfungsfragen zwischen den unterschiedlichen Lernplattformen sowie die Optionen für die Durchführung paralleler Online-Prüfungen auf den Plattformen müsste in einem nächsten Schritt genauer geprüft werden.

Zum anderen lassen sich die kuratierten Lehr- und Prüfmaterialien auch für weitere Iterationen der Lehrveranstaltung wiederverwenden, erweitern und mit weiteren interessierten Hochschulen austauschen. Dieses Vorgehen ist in Nordrhein-Westfalen (NRW) bereits unter dem Dach der „Digitalen Hochschule NRW“ im Aufbau. Auf einem Online-Landesportal sollen u. a. frei zugängliche Lehr- und Lernmaterialien für Lehrende und Studierende bereitgestellt werden (Open Resources Campus NRW). Langfristig wäre damit auch eine komplette Bereitstellung der im Kontext der beschriebenen Lehrveranstaltung erstellten Materialien auf dem Landesportal denkbar. Um auf einer landesweiten Austauschplattform Materialien zur Verfügung zu stellen und zu nutzen, ist die Qualitätssicherung der Materialien in einem vertrauensvollen Umfeld sicherzustellen. Ist dies gewährleistet, kann ein solches Online-Portal hochschulformübergreifenden Kollaboration sowie den Austausch und die gemeinsame Nutzung von Lehr- und Lernmaterialien unterstützen.
